# Data acquisition for the UK Critical Care Minimum Data Set: validation of a computer model for automatic calculation from an electronic patient record

**DOI:** 10.1186/cc11117

**Published:** 2012-03-20

**Authors:** A Clarke, M Thomas, T Gould, C Bourdeaux

**Affiliations:** 1 Bristol Royal Infirmary, Bristol, UK

## Introduction

This study reports the accuracy of a computer and a manual system at collecting data for the UK Critical Care Minimum Data Set (CCMDS). This is required by the Department of Health to compare performance, to facilitate funding and to plan future resource provision. There are 14 data fields in the mandatory dataset, and the full compliment extends to 34 fields. At present this is collected manually, which is laborious and subjective. We use an electronic patient record (Innovian, Draeger, Germany) to store all the measured patient observations and laboratory results. We have written a program to interrogate Innovian for the CCMDS data, thereby reducing the administrative time.

## Methods

A stratified sample of 50 patients' data (elective and emergency surgical and medical patients) was analysed. Both manual and computer systems collected the mandatory 14 items of the CCMDS. This consists of six demographic variables (for example, admission date, discharge date, date of birth) and eight organ support variables (for example, duration of either advanced or basic cardiovascular, respiratory, renal or neurological support or duration of level 2 or 3 support). Where the computer and manual systems returned different values, a blinded physician analysed the patient records and created a gold standard value. The frequency of these differences was analysed.

## Results

Both computer and manual systems returned all the required data, giving a total of 700 data variables. Different values were returned for 183 (26%) variables. The systems had good concordance in the demographic variables, with only 4/300 (1.3%) discrepancies between the computer and manual systems. In the organ support variables, there were 179/400 (45%) discrepancies. Days of renal support had most concordance, with discrepancies in 3/50 patients (6%). Days of level 2 support had least concordance, with discrepancies in 37/50 patients (76%). Overall, the computer method returned the correct variable for 544 (78%) variables, where the manual system returned the correct variable on 591 (84%) variables.

## Conclusion

This study shows that both computer and manual data collection methods could be improved, but at present both have similar accuracy. This may be because the criteria for some organ support can be subjective (for example, risk of deterioration), which can be interpreted in different ways between manual data collectors but not by a computer. We plan to rewrite the computer program, aiming for >95% concordance with the gold standard.

